# The First Mitogenome of the Cyprus Mouflon (*Ovis gmelini ophion*): New Insights into the Phylogeny of the Genus *Ovis*

**DOI:** 10.1371/journal.pone.0144257

**Published:** 2015-12-04

**Authors:** Daria Sanna, Mario Barbato, Eleftherios Hadjisterkotis, Piero Cossu, Luca Decandia, Sandro Trova, Monica Pirastru, Giovanni Giuseppe Leoni, Salvatore Naitana, Paolo Francalacci, Bruno Masala, Laura Manca, Paolo Mereu

**Affiliations:** 1 Dipartimento di Scienze della Natura e del Territorio, University of Sassari, Sassari, Italy; 2 Cardiff School of Biosciences, Cardiff University, Cardiff, United Kingdom; 3 Ministry of the Interior, Nicosia, Cyprus; 4 Department of Biomedical Sciences, University of Sassari, Sassari, Italy; 5 Department of Veterinary Medicine, University of Sassari, Sassari, Italy; 6 Center of Excellence for Biotechnology Development and Research on Biodiversity, University of Sassari, Sassari, Italy; University of Perugia, ITALY

## Abstract

Sheep are thought to have been one of the first livestock to be domesticated in the Near East, thus playing an important role in human history. The current whole mitochondrial genome phylogeny for the genus *Ovis* is based on: the five main domestic haplogroups occurring among sheep (*O*. *aries*), along with molecular data from two wild European mouflons, three urials, and one argali. With the aim to shed some further light on the phylogenetic relationship within this genus, the first complete mitochondrial genome sequence of a Cypriot mouflon (*O*. *gmelini ophion*) is here reported. Phylogenetic analyses were performed using a dataset of whole *Ovis* mitogenomes as well as *D-loop* sequences. The concatenated sequence of 28 mitochondrial genes of one Cypriot mouflon, and the *D-loop* sequence of three Cypriot mouflons were compared to sequences obtained from samples representatives of the five domestic sheep haplogroups along with samples of the extant wild and feral sheep. The sample included also individuals from the Mediterranean islands of Sardinia and Corsica hosting remnants of the first wave of domestication that likely went then back to feral life. The divergence time between branches in the phylogenetic tree has been calculated using seven different calibration points by means of Bayesian and Maximum Likelihood inferences. Results suggest that urial (*O*. *vignei*) and argali (*O*. *ammon*) diverged from domestic sheep about 0.89 and 1.11 million years ago (*MYA*), respectively; and dates the earliest radiation of domestic sheep common ancestor at around 0.3 *MYA*. Additionally, our data suggest that the rise of the modern sheep haplogroups happened in the span of time between six and 32 thousand years ago (*KYA*). A close phylogenetic relationship between the Cypriot and the Anatolian mouflon carrying the X haplotype was detected. The genetic distance between this group and the other ovine haplogroups supports the hypothesis that it may be a new haplogroup never described before. Furthermore, the updated phylogenetic tree presented in this study determines a finer classification of ovine species and may help to classify more accurately new mitogenomes within the established haplogroups so far identified.

## Introduction

The knowledge of species origin has always been an essential element for informed genetic diversity conservation. In particular, the evolution of domestic species has been intensively investigated during the last few decades. According to Vigne [1], archaeozoological findings suggest that the earliest detected domestications occurred in the Near East during the 11^th^ millennium before present (*BP*). In such a context, sheep and goats were the first livestock to be domesticated near the region known as Fertile Crescent [2]. In this area, the domestication of the Asian mouflon (*Ovis gmelini*) probably gave rise to the domestic sheep (*O*. *aries*) [1, 3–7], although the contribution of other wild species such as urial (*O*. *vignei*), argali (*O*. *ammon*) and European mouflon (*O*. *a*. *musimon*) has been suggested [8–10].

To date, five sheep haplogroups (*HPGs*) have been identified, including the last discovered *HPG* E [11]. The latter is the rarest haplogroup together with *HPG* D, whereas *HPG* C is the third more widespread, with samples retrieved in Asia, Fertile Crescent, Caucasus and the Iberian Peninsula. *HPGs* A and B are the most common ovine haplogroups; the first being more recurrent in Middle East, Asia, and Europe, while *HPG* B in European sheep [11]. Furthermore, molecular investigations based on the analysis of a partial sequence of the mitochondrial *D-loop* region [12] supported the occurrence of a new ovine haplotype, never observed before among domestic sheep; this was detected in Anatolian mouflons whose gene pool is composed of two different *mtDNA* haplotypes, one belonging to *HPG* A and one closely related to *HPGs* C and E, named by the authors as haplotype X.

Analyses based on comparisons of the whole *mtDNA* sequence, which significantly improve the resolution power of phylogenetic analyses if compared with single genes or small *DNA* fragments, clearly confirmed that neither urial nor argali sheep are the maternal ancestor of the domestic sheep [13]. In addition, the European mouflon should not be considered a truly wild sheep; instead, it more likely represents a remnant from early domestication events which has readapted to feral life [14–16].

Some authors described the Asian mouflon (*O*. *gmelini*) and the urial (*O*. *vignei*) as a single “moufloniform” species (*O*. *orientalis*) [17–18]; instead, the classification that distinguishes the Asian mouflon from urial was followed in this study [14, 19].

Currently, the revised taxonomy of *O*. *gmelini* [20] differentiates the taxa into several “subspecies”. The Armenian mouflon (*O*. *g*. *gmelinii*) from western Iran and easternmost Turkey, is considered the most probable ancestor of domestic sheep along with the Anatolian mouflon (*O*. *g*. *anatolica*) endemic to central Anatolia. A third subspecies has been assigned to the Cyprus mouflon (*O*. *g*. *ophion*), also known as agrino, a wild sheep found exclusively in the Mediterranean island of Cyprus. The agrino is the only wild representative of the Caprinae subfamily on the island, and the largest animal of the local wild fauna [21]. The mouflon population in Cyprus currently counts ~3,000 individuals living in the mountainous area of the Paphos forest, a region of 620,000 hectares located in the North West of the island and classified as a Special Protection Area since 2005.

The *IUCN* [22] included the Cypriot mouflon in the list of species considered as "vulnerable" due to poaching, habitat loss and fragmentation caused by the presence of road networks, and infection by pathogens (see [23] for details). To make matters worse, climate models predict that climate change will lead to higher temperatures and less rainfall in the Eastern Mediterranean with deleterious impact on the agricultural system and animal health [24].

The presence of mouflon in Cyprus is believed to date back to around 10,000 years ago (*YA*) [2]. Recent paleontological findings [25] indicated that the first sheep (*O*. *aries*) were introduced in Cyprus during the first half of the 10^th^ millennium *BP*, then replaced 500 years later by bigger sheep presumably introduced in the island from Syria. Based on the shape and size of the horns of these prehistoric animals, these sheep were similar to the wild sheep of the nearby mainland [25–26].

To date, only small portions of the *mtDNA* and nuclear *DNA* of the Cyprus mouflon are available. These segments include the complete sequence of the mitochondrial Cytochrome b gene (*Cyt B*) (*GB*#FR873149) [23], and the entire sequence of the alpha 1 (*GB*#EU938070.1), alpha 2 (*GB*#EU938071) [27] and beta (*GB*#DQ352469) [28] nuclear globin genes. Based on partial *Cyt B* sequence analyses, *O*. *g*. *ophion* clustered with the ovine *HPG*s E and C, whereas its *D-loop* belonged to *HPG* B [12, 29].

The aim of this study was to shed some light on the evolutionary pathway that led to the emergence of current sheep haplogroups including as a new resource the Cyprus mouflon mitogenome. Mitochondrial *DNA* was used to infer evolutionary history and phylogeographic relationships among current species of the *Ovis* genus. In such a context, 27 mitogenome sequences were used to obtain a whole *mtDNA* genome-based phylogenetic tree of *Ovis* species. Such analysis was combined to a molecular dating to infer the evolutionary divergence timing among *Ovis* species providing a possible coalescence time for the rise of the current sheep haplogroups. To achieve a more comprehensive picture of the phylogenetic relationships among ovine haplogroups we analysed 35 additional *D-loop* sequences. The *D-loop* of three agrinos were sequenced and compared with the homologous region from representative samples of the five ovine haplogroups identified so far.

Animal species domestication has been carried out through the cross-breeding persistence with wild population [30], including Cyprus mouflon. This process started after the last glaciations when the increasing temperature determined abrupt environmental changes with the extinction of some species and the success of others [31]. The Cyprus mouflon would well represent the species which adapted to new environment. The present study, providing new information on the genetics of the Cyprus mouflon, would help to improve the efficiency of selection on breed traits linked to reproductive performance and to maintain good productive skills in arid environments.

## Materials and Methods

The Ethics Committee of the University of Sassari, Italy, approved this study.

Peripheral blood samples were obtained from five Sardinian, three Cypriot and two Corsican mouflons and two Sardinian and two Chios sheep. The dataset was implemented downloading from Genbank 26 whole mitogenomes and 21 *D-loop* sequences (see Table 1 for details).

**Table 1:** List of the species and the sequences included in phylogenetic analyses.

**a) Whole mitogenome (*28H*)**					
**Scientific name**	Common name	*HPG*	Code	Geographic origin	*GB* #
***Addax nasomaculatus***	White screwhorn antelope	-	-	-	NC_020674
***Bos taurus***	Cattle	-	-	-	NC_006853
***Boselaphus tragocamelus***	Nilgai antelope	-	-	-	NC_020614
***Bubalus bubalis***	Water buffalo	-	-	-	AY488491
***Connochaetes taurinus***	Blue wildebeest	-	-	-	NC_020699
***Hippotragus niger***	Sable antelope	-	-	-	NC_020713
***Kobus ellipsiprymnus***	Waterbuck	-	-	-	NC_020715
***Kobus leche***	Red lechwe	-	-	-	NC_018603
***Moschus moschiferus***	Siberian musk deer	-	-	-	JN632662
***Oreamnos americanus***	Rocky Mountain goat	-	-	-	FJ207535
***Pantholops hodgsoni***	Tibetan antelope	-	-	-	DQ191826
***Redunca arundinum***	Southern reedbuck	-	-	-	NC_020794
***Ovis ammon***	Argali	-	AWS	Kazakhstan	HM236188
***Ovis aries***	Domestic sheep	A	RA_1	Australia	HM236174
***Ovis aries***	Domestic sheep	A	RA_2	Australia	HM236175
***Ovis aries***	Domestic sheep	B	RB_1	Turkey	HM236176
***Ovis aries***	Domestic sheep	B	RB_2	Turkey	HM236177
***Ovis aries***	Domestic sheep	C	RC_1	Turkey	HM236178
***Ovis aries***	Domestic sheep	C	RC_2	Turkey	HM236179
***Ovis aries***	Domestic sheep	D	RD_1	Turkey	HM236180
***Ovis aries***	Domestic sheep	D	RD_2	Turkey	HM236181
***Ovis aries***	Domestic sheep	E	RE_1	Israel	HM236182
***Ovis aries***	Domestic sheep	E	RE_2	Turkey	HM236183
***Ovis aries musimon***	European mouflon	B	EUM	Germany	HM236184
***Ovis canadensis***	Bighorn	-	BWS	Canada	JN181255
***Ovis gmelini ophion***	Cyprus mouflon	-	CYM	Cyprus	KF312238*
***Ovis vignei***	Urial	-	UWS	Kazakhstan	HM236189
**b) *D-loop***					
**Scientific name**	Common name	*HPG*	Code		*GB*#
***Ovis aries***	Domestic sheep	A	RA_1	Turkey	DQ852286
***Ovis aries***	Domestic sheep	A	RA_2	Turkey	DQ852287
***Ovis aries***	Domestic sheep	B	RB_1	Turkey	DQ852282
***Ovis aries***	Domestic sheep	B	RB_2	Turkey	DQ852285
***Ovis aries***	Domestic sheep	C	RC_1	Turkey	DQ852284
***Ovis aries***	Domestic sheep	C	RC_2	Turkey	DQ852283
***Ovis aries***	Domestic sheep	D	RD_1	Turkey	DQ852288
***Ovis aries***	Domestic sheep	D	RD_2	Turkey	DQ852289
***Ovis aries***	Domestic sheep	E	RE_1	Israel	DQ852280
***Ovis aries***	Domestic sheep	E	RE_2	Israel	DQ852281
***Ovis aries***	Chios sheep	B^&^	CHS_1	Chios	KR011777*
***Ovis aries***	Chios sheep	B^&^	CHS_2	Chios	KR011778*
***Ovis aries***	Sardinian sheep	B^&^	SAS_1	Sardinia	KR011770*
***Ovis aries***	Sardinian sheep	B^&^	SAS_2	Sardinia	KR011771*
***Ovis aries musimon***	European mouflon	B^&^	EUM_1	Germany	HM236184
***Ovis aries musimon***	European mouflon	B^&^	EUM_2	Germany	HM236185
***Ovis aries musimon***	Sardinian mouflon	B^&^	SAM_1	Sardinia	KR011772*
***Ovis aries musimon***	Sardinian mouflon	B^&^	SAM_2	Sardinia	KR011773*
***Ovis aries musimon***	Sardinian mouflon	B^&^	SAM_3	Sardinia	KR011774*
***Ovis aries musimon***	Sardinian mouflon	B^&^	SAM_4	Sardinia	KR011775*
***Ovis aries musimon***	Sardinian mouflon	B^&^	SAM_5	Sardinia	KR011776*
***Ovis aries musimon***	Corsican mouflon	B^&^	COM_1	Corsica	KR011781*
***Ovis aries musimon***	Corsican mouflon	B^&^	COM_2	Corsica	KR011782*
***Ovis gmelini ophion***	Cyprus mouflon	-	CYM_1	Cyprus	KR011779*
***Ovis gmelini ophion***	Cyprus mouflon	-	CYM_2	Cyprus	KF312238*
***Ovis gmelini ophion***	Cyprus mouflon	-	CYM_3	Cyprus	KR011780*
***Ovis gmelini anatolica***	Anatolian mouflon	X^$^	ANM_1	Turkey	KF677264
***Ovis gmelini anatolica***	Anatolian mouflon	X^$^	ANM_2	Turkey	KF677265
***Ovis gmelini anatolica***	Anatolian mouflon	X^$^	ANM_3	Turkey	KF677266
***Ovis gmelini anatolica***	Anatolian mouflon	A^&^	ANM_4	Turkey	KF677267
***Ovis gmelini anatolica***	Anatolian mouflon	A^&^	ANM_5	Turkey	KF677268
***Ovis gmelini anatolica***	Anatolian mouflon	A^&^	ANM_6	Turkey	KF677269
***Ovis ammon***	Argali wild sheep	-	AWS	Kazakhstan	HM236188
***Ovis canadensis***	Bighorn wild sheep	-	BWS	Canada	JN181255
***Ovis vignei***	Urial wild sheep	-	UWS	Kazakhstan	HM236189

*HPG*: haplogroup; *GB*#: Genbank accession number.

^$^ X for the Anatolian mouflon *O*. *g*. *anatolica* represents an haplotype whose haplogroup was not described by Demirci et al.[12].

* Sequences obtained in the present study.

^&^ *HPG* inferred by phylogenetic analysis.

Furthermore, the data matrix included ten *mtDNA* whole genome sequences from sheep representing the five main mitochondrial haplogroups (A, B, C, D, E) (two sequences per haplogroup). The water buffalo [32], the Tibetan antelope [33], the Rocky Mountain goat [34], the Southern antelope, the white screwhorn antelope, the nilgai antelope, the blue wildebeest, the waterbuck, the red lechwe, the Siberian musk deer [35] and the cattle sequences were used as outgroups for the phylogenetic tree analysis (see Table 1 for scientific names and Genbank accession numbers).

Genomic *DNA* was extracted using the GenElute blood genomic DNA kit (Sigma-Aldrich) according to the manufacturer's protocol. Sample quality and *DNA* concentration were determined via spectrophotometry using a ND-8000 (NanoDrop Technologies, Thermo Fisher Scientific Inc., Wilmington, DE). The *DNA* mean concentration obtained was 125 ng/μL.

### Mitogenome amplification and sequencing

To perform amplification experiments, specific primers were designed for the most conserved regions belonging to all *Ovis* species mitogenomes available in databases.

A primer pair was selected when the average size of the amplicon was 1,100 base pairs (*bp*) long to allow sequencing reactions by means of the same primer. Each adjacent fragment had at least 100 *bp* of overlap to ensure complete sequencing coverage and the average annealing temperature was approximately 56°C.

A total of 21 primer pairs were selected with an average overlapping of 212 *bp* long fragments; the amplifications involved also two nested *PCR* reactions (see S1 Table for details).

*PCR* and sequencing reaction were performed according to the protocols provided by Pirastru et al. [27] and Manca et al. [28]. The annealing conditions for each primer are specified in S1 Table.

The failure of the sequencing reaction for some amplicons, required the design of 5 additional sequencing primers to complete the whole mitogenome sequence (S1 Table).

Raw sequencing data were processed by Sequencing Analysis Software 5.3.1 (Applied Biosystem) and the quality value of each base in the electropherograms was assessed by the KB base-calling algorithm. Processed sequences were visualized using FinchTV 1.4.0 (Geospiza Inc.) and assembled into contigs, after identifying overlapping areas on Clustal X 2 [36]. Gene arrangement was identified by comparing the sequences to that of *O*. *aries* (*GB*# NC_001941). Double peaks of similar height, which may be interpreted as evidence of mitochondrial pseudogenes in the nucleus (*Numts*) or heteroplasmy, were not observed in any of the electropherograms.

Protein coding genes were detected by means of ORF Finder software (http://www.ncbi.nlm.nih.gov/gorf.html) by setting the type of mitochondrial code to the vertebrate genetic code. The *tRNA* genes were identified by the online program tRNA-scan SE (http://lowelab.ucsc.edu/tRNAscan-SE) using the mito/chloroplast genetic code and the default search mode.

**Fig 1:**
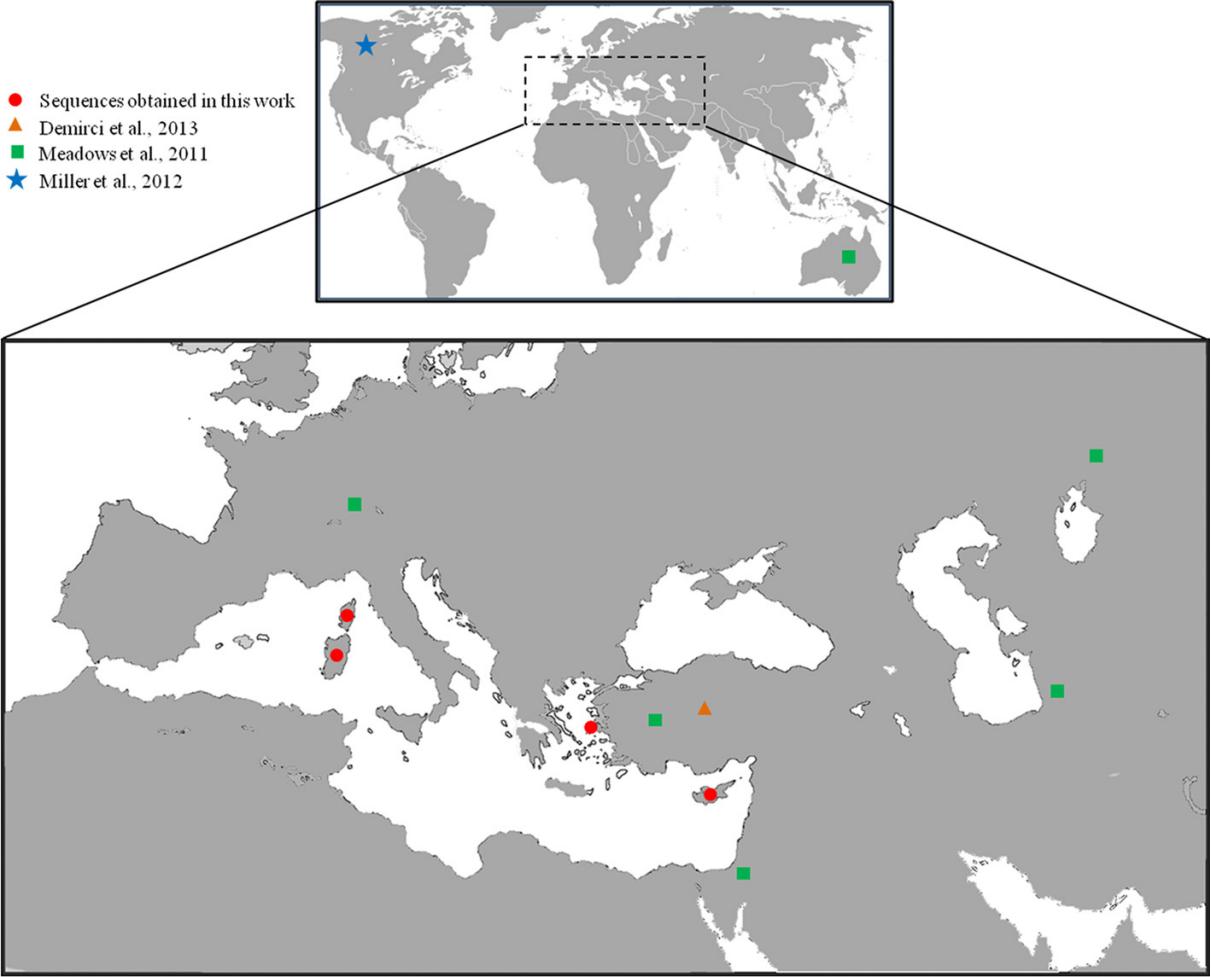
Sampling plan. World map with enlargement on the Mediterranean basin, indicating the geographical distribution of samples used in the present study.

In order to assess the number and the length of tandem repeats in the control region, which differentiate ovine haplogroups, the *D-loop* sequences were tested using Tandem Repeat Finder 3.01 [37]. The physical map of the Cyprus mouflon mitogenome was generated by means of OGDraw 1.2 [38]. The geographical distribution of sheep samples analyzed in the present study is shown in Fig 1.

### Phylogenetic analysis of whole genome

In order to carry out phylogenetic analysis in accordance with a clock-like model we used the concatenated sequences of the 12 protein-coding genes, 14 transfer *RNA* genes and two ribosomal *RNA* genes, all located on the H-strand. Hereafter, we will refer to these mitochondrial segments as a unique molecular marker called *28H*. The *D-loop* sequence was analysed as a single marker due to its higher nucleotide substitution frequency. Additionally, mutations are not randomly distributed across the length of the locus, making rate heterogeneity an important issue in calculating divergence date estimates [39–41].

Sequences were aligned using the programme CLUSTAL W [42], implemented in the BioEdit 7.0.5.2 software package [43]. The genetic variation within the genus *Ovis* was assessed estimating the number of polymorphic sites (S), number of haplotypes (H), haplotype diversity (h), and nucleotide diversity (π) using the software package DnaSP 5.10 [44].

MEGA 6.06 [45] was used to choose the nucleotide substitution model. According to the lowest BIC scores (Bayesian Information Criterion), *AICc* value (Akaike Information Criterion, corrected), Maximum Likelihood value (*lnL*), and the number of parameters, the general time-reversible (*GTR*) [46] was selected for the three codon positions of the dataset. Non-uniformity of evolutionary rates among sites was modeled by using a discrete Gamma distribution (+*G*) with 6 rate categories and by assuming that a certain fraction of sites are evolutionarily invariable (+*I*).

Phylogenetic relationships among individuals were investigated by means of Bayesian Inference (*BI*) and Maximum Likelihood (*ML*) analysis using the *28H* segment. MrBayes 3.2.4 [47] was used for *BI*. In order to search for the optimal evolutionary model combination for each gene, the *28H* region was split up in 28 different segments corresponding to the mitochondrial genes that compose it. Two independent runs, each consisting of four Metropolis-coupled reversible-jump Markov chain Monte Carlo chains were performed. A Dirichlet distribution with quasi-flat priors (1, 2, 1, 1, 2, 1) was assumed for estimating the substitution frequency parameters. Analyses were carried out for 5 million generations and the trees were sampled every 10 generations. Convergence of chains was checked by ensuring that the standard deviation of split frequencies reached and stabilized at a value < 0.01 and by verifying the stationarity of the generations/log probability graph [48]. For each run, the first 25% sampled trees were discarded.

A *ML* tree reconstruction was performed using a *GTR* model by TREEPUZZLE 5.2 [49]. *A priori* tests for the detection of a phylogenetic signal were performed using the likelihood mapping option and the reliability of each branch was estimated by bootstrapping (10,000 puzzling steps). Molecular dating was carried out assuming seven different calibration points (*CPs*) based on fossil records that provide ages for nodes inside Bovidae [50] (see Table 2 for details).

**Table 2:** Calibration points used for molecular datings.

Calibrated node/branch	Name	Age type	95% range (*MYA*)
Crown *Kobus*	CP-1	Minimum	2.0–3.0
Crown Hippotragini	CP-2	Minimum	3.6–6.5
Crown Reduncini	CP-3	Minimum	5.1–7.0
Stem Hippotragini	CP-4	Minimum	6.4–13.0
Stem Caprini	CP-5	Minimum	8.9–13.0
Stem Bovini	CP-6	Minimum	10.2–16.0
Crown Bovidae	CP-7	Approximate	16.0–20.0

*MYA*: million years ago.

Estimates of divergence times were obtained using the Bayesian approach implemented in BEAST 1.7.5 [51].

Sequences were analysed under the *GTR*+*G*+*I* model of sequence evolution, with 4 gamma categories, a Yule process speciation rate, and empirical base frequencies. We assumed a lognormal relaxed molecular clock with uncorrelated rates [51], setting the priors for multiple calibration points used in this study (Table 2) as described in Bibi et al. [50]. Four independent runs were carried out with the following settings: 20,000,000 steps, drawing data to file every 2,000 steps in order to obtain 10,000 records and trees. The output of two independent runs was analysed using Tracer 1.5 [52], applying a post-processing burn-in of 10% thus discarding the first 1,000 records for each run. Analysis of log files indicated for all parameters convergence of independent runs and the combined effective sample size (*ESS*) was >200. Subsequently, a maximum clade credibility (*MCC*) tree was created by (i) using a logcombiner [51] to merge tree files from each independent run after removing 10% of initial trees as burn-in and subsequently resampling of states to obtain a final sample of 9,000 trees, and (ii) using a tree annotator to create the consensus tree.

Time to the most recent common ancestors (*TMRCAs*) was also estimated using two other methods based on the Maximum Likelihood framework. The first one is the RelTime method implemented in MEGA 6.06 and does not assume any specific lineage rate of evolution [45]. Divergence times for all branching points in the topology were calculated using Maximum Likelihood based on *GTR*+*G*+*I* model, and allowing for all the possible local clocks. 95% confidence intervals around each estimate were computed following the method of Tamura et al. [42]. With the second method, which is implemented in the APE R-package (R Core Team 2015) [53], *TMRCAs* was estimated using the Penalised Likelihood and Maximum Likelihood algorithm developed by Paradis et al. [54]. The algorithm is an improvement of the penalized likelihood method developed by Sanderson [55] and is available in the function Chronos. Divergence times were estimated assuming the strict model for substitution rates along the tree and setting the smoothing parameter to the default value.

### Phylogenetic analysis of *D-loop* region

The phylogenetic analysis of the *mtDNA* control region was performed on a dataset including 35 *Ovis* sequences (21 from wild and 14 from domestic sheep), 14 of which were obtained in this study (see Table 1B for details). Sequences were aligned and the genetic variation was assessed as above reported.

The data matrix, including ten sheep *D-loop* sequences representing the five main mitochondrial haplogroups (two per each *HPG*), was used to carry out both a Bayesian and Maximum Likelihood analyses according to methodologies above reported.

Due to the larger number of sequences available for this marker, the presence of a genetic structure among haplogroups was also assayed by the Bayesian model-based clustering algorithm implemented in BAPS 5.3 [56]. Clustering was performed using the module for linked molecular data and applying the codon linkage model, which is appropriate for sequencing data. The analysis was run ten times with a vector of K values = 2 to 22, each with six replicates. Haplotypes were organized into haplogroups according to the genetic structure evidenced by Bayesian clustering.

A 95% statistical parsimony network analysis was performed using the software package TCS 1.21 [57], aimed at searching for possible disconnections between groups of individuals, further inferring the genetic relationships among the haplotypes. Gaps were treated as a fifth character state.

Statistical parsimony is an alternative method for network construction that joins haplotypes within a parsimony connection limit, the latter being the maximum number of differences not due to reversion between haplotypes for which a 95% confidence exists.

The software package Network 4.5.0.1 (www.fluxus-engineering.com) was used to construct a median-joining network [58] to be superimposed on the sample map in order to infer the genetic relationships among the haplotypes and analyze the occurrence of discrete geographic genetic clusters. Transitions and transversions were equally weighted (default option).

The pairwise genetic distances corrected according to the Kimura two-parameter model (*K2P*) [59] were estimated between individuals by means of the software MEGA 6.06 [45] with 1,000 bootstrap replicates.

## Results

**Fig 2:**
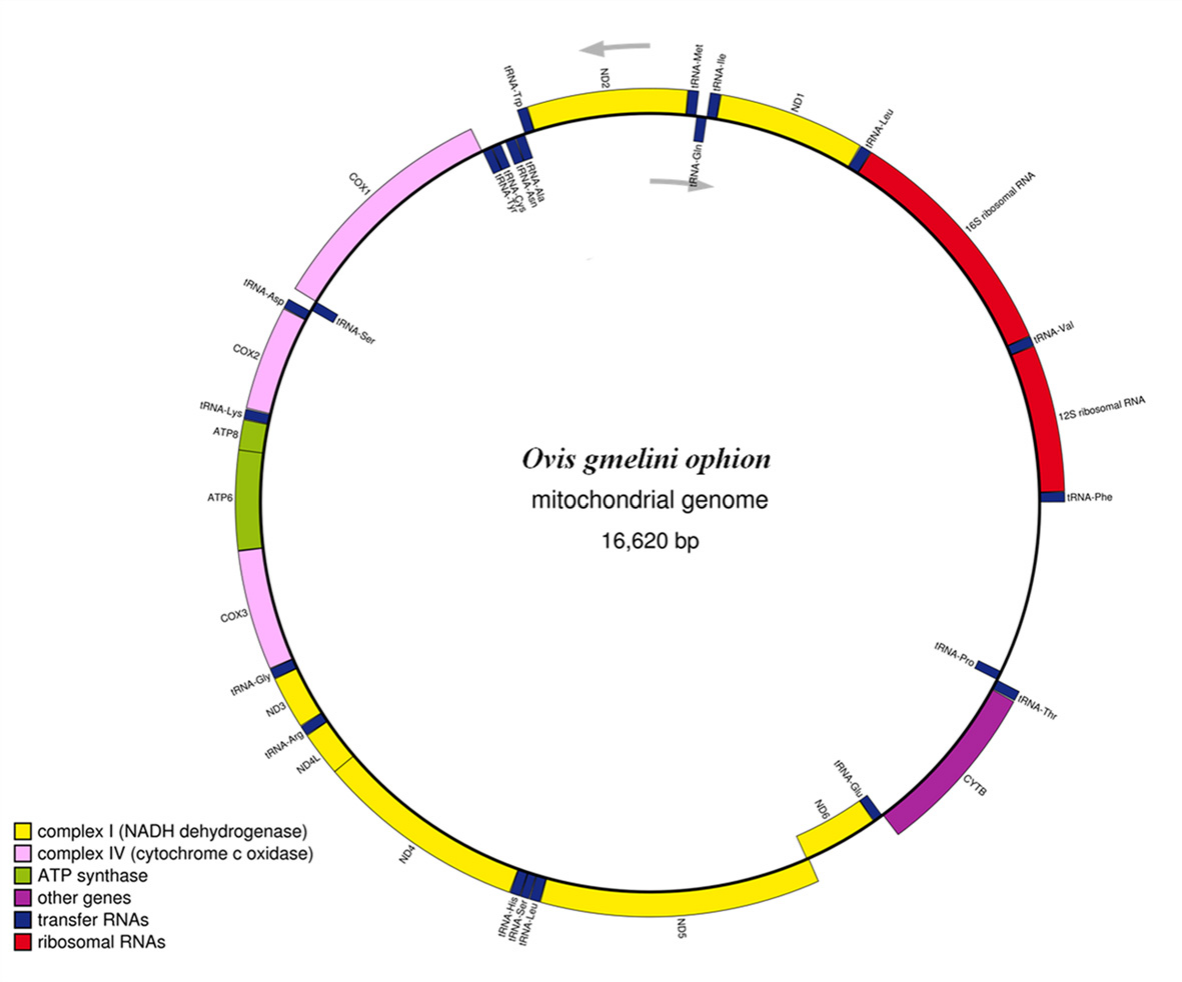
Structural organization of Cyprus mouflon mitogenome. Arrows indicate the reading frame orientation of each strand.

We determined the complete nucleotide sequence of the mitogenome of the Cyprus mouflon (*GB*# KF312238) (Fig 2).

The *mtDNA* molecule of *O*. *g*. *ophion* was 16,620 *bp* long, very similar in length to what was found for the domestic sheep (16,616 *bp*). Analyzing both the H and L strand 13 protein coding genes, *12S* and *16S* ribosomal *RNA*, 22 transfer *RNA* genes and a *D-loop* were identified. Locations of the various features and the molecule gene content are displayed in Fig 2 and summarized in Table 3, together with the inferred start and stop codons as determined by comparison with the homologous gene sequences of domestic sheep.

**Table 3:** Organization of the Cyprus mouflon mitochondrial genome.

Gene	Location	Size	Start codon	Stop codon	3’ spacer/overlap
*tRNA*-Phe	1	68			
*12S rRNA*	69	958			
*tRNA*-Val	1,027	67			
*16S rRNA*	1,094	1,575			
*tRNA*-Leu	2,669	75			AA-base spacer
*NADH* 1	2,746	957	ATG	TAA	1-base overlap
*tRNA*-Ile	3,702	69			3-base overlap
*tRNA*-Gln (L)	3,768	72			AT-base spacer
*tRNA*-Met	3,842	69			
*NADH* 2	3,911	1,042	ATA	Taa*	
*tRNA*-Trp	4,953	67			A-base spacer
*tRNA*-Ala (L)	5,021	69			A-base spacer
*tRNA*-Asn (L)	5,091	73			
*O*_*L*_	5,164	32			
*tRNA*-Cys (L)	5,196	68			
*tRNA*-Tyr (L)	5,264	68			C-base spacer
*COX* I	5,333	1,545	ATG	TAA	3-base overlap
*tRNA*-Ser (L)	6,875	71			AAC-base spacer
*tRNA*-Asp	6,951	68			T-base spacer
*COX* II	7,020	684	ATG	TAA	AAT-base spacer
*tRNA*-Lys	7,707	68			T-base spacer
A*T*P 8	7,776	201	ATG	TAA	40-base overlap
*ATP* 6	7,937	681	ATG	TAA	1-base overlap
*COX* III	8,617	784	ATG	Taa*	
*tRNA*-Gly	9,401	69			
*NADH* 3	9,470	347	ATA	TAa*	T-base spacer
*tRNA*-Arg	9,818	68			
*NADH* 4L	9,886	297	ATG	TAA	7-base overlap
*NADH* 4	10,176	1,378	ATG	Taa*	
*tRNA*-His	11,554	69			
*tRNA*-Ser	11,623	60			A-base spacer
tR*N*A-Leu	11,684	70			
*NADH* 5	11,754	1,821	ATA	TAA	17-base overlap
*NADH* 6 (L)	13,558	528	ATG	TAA	
*tRNA*-Glu (L)	14,086	69			ACTA-base spacer
*Cyt B*	14,159	1,140	ATG	AGA	CAA-base spacer
*tRNA*-Thr	15,302	70			1-base overlap
*tRNA*-Pro (L)	15,371	66			
*D-loop*	15,436	1,184			

In the Gene column (L) indicates a gene encoded on the L-strand.

* Incomplete stop signals.

The percentage composition in bases of the L-strand is 33.6% A, 25.8% C, 13.1% G, and 27.4% T, in accordance with those obtained for *O*. *aries* [60]. As commonly observed in mammal mitogenomes, *ND6* and eight *tRNA* genes are encoded in the L-strand, and all protein-coding genes have the ATG start codon, except for *ND2* and *ND3* genes, that begin with ATA. *Cyt B* is the only gene that stops with AGA instead of the TAA codon which is incomplete in the *ND2*, *ND4*, *COIII* (T) and *ND3* (TA) genes. The origin of the light strand replication (*O*_*L*_) is located at the 5,164–5,195 nt region, and has the same sequence as the domestic sheep [60].

The *mtDNA* sequence of the Cyprus mouflon matches with those of other members of the Caprinae sub-family, and features like incomplete stop codons, overlapping coding regions, and different start codons lie within the range of mammalian *mtDNA* variation [60–61].

### Whole genome (*28H*): phylogenetic analyses and molecular dating

The complete Cyprus mouflon *mtDNA* sequence was compared with the homologous sequences of the five sheep *mtDNA* haplogroups identified by Meadows et al. [13] (see Table 1 for details).

**Fig 3:**
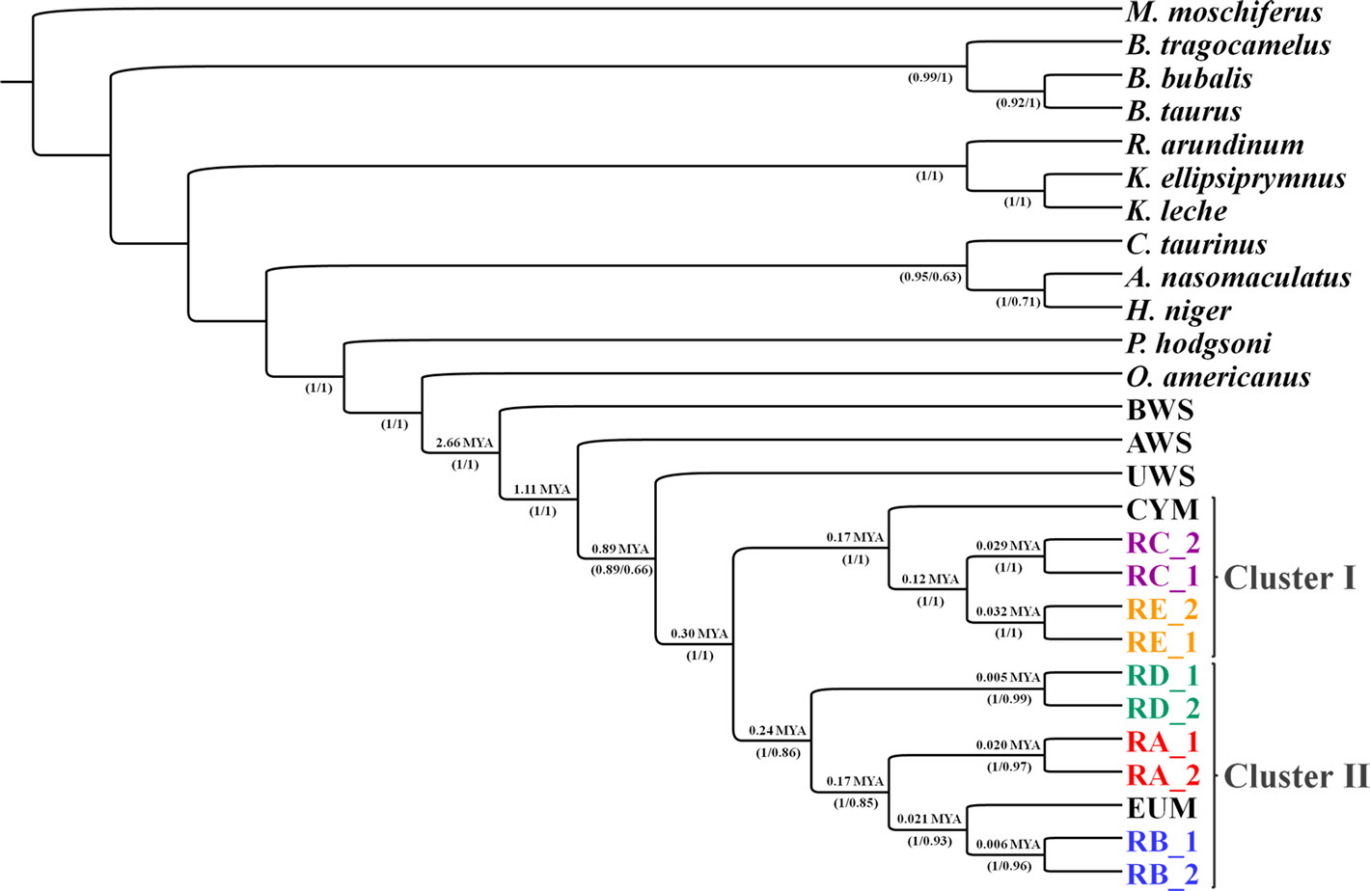
Rooted tree obtained by Bayesian inference for *28H* dataset showing two clusters of sheep haplogroups. Nodal supports are indicated below the nodes (posterior probability for *BI* / bootstrap values for *ML*). Molecular dating in million years are indicated above the nodes. Sample codes are listed in Table 1.

Overall, 27 sequences representative of bovid species were included into the dataset and the *M*. *moschiferus* sequence was used as outgroup (Fig 3).

Among 15 individuals belonging to six species within the *Ovis* genus, 15 haplotypes, defined by 1,094 polymorphic sites (S), were found. Overall, high values of total mean haplotype and nucleotide diversity, were obtained (h = 1 and π = 0.016, respectively). A lower value of nucleotide diversity (π = 0.006) was found among the individuals of the species *O*. *aries*. Estimates of genetic diversity for the whole genome are reported in Table 4.

**Table 4:** Genetic diversity estimates obtained for whole genome (*28H*) (a) and *D-loop* (b) datasets.

**a) Whole mitogenome (*28H*) 14,426 *bp***						
**Scientific name**	Common name	N	S	h	H	π
***Ovis ammon***	Argali	1	0	1	0	0
***Ovis aries***	Domestic sheep	10	229	10	1	0.006
***Ovis aries musimon***	European mouflon	1	0	1	0	0
***Ovis canadensis***	Bighorn	1	0	1	0	0
***Ovis gmelini ophion***	Cyprus mouflon	1	0	1	0	0
***Ovis vignei***	Urial	1	0	1	0	0
**TOT**		15	1,094	15	1	0.016
**b) *D-loop* 1,160 *bp***						
**Scientific name**	Common name	N	S	h	H	π
***Ovis aries***	Domestic sheep	10	82	6	0.911	0.029
***Ovis aries***	Chios sheep	2	16	2	1	0.014
***Ovis aries***	Sardinian sheep	2	10	2	1	0.009
**TOT**		14	99	10	0.956	0.027
***Ovis aries musimon***	European mouflon	2	0	1	0	0
***Ovis aries musimon***	Sardinian mouflon	5	14	4	0.900	0.006
***Ovis aries musimon***	Corsican mouflon	2	6	2	1	0.005
**TOT**		9	29	7	0.944	0.009
***Ovis gmelini ophion***	Cyprus mouflon	3	7	3	1	0.004
**TOT**		3	7	3	1	0.004
***Ovis gmelini anatolica***	Anatolian mouflon	6	42	2	0.600	0.023
**TOT**		6	42	2	0.600	0.023
***Ovis ammon***	Argali wild sheep	1	0	1	0	0
***Ovis canadensis***	Bighorn wild sheep	1	0	1	0	0
***Ovis vignei***	Urial wild sheep	1	0	1	0	0
**TOT**		35	645	23	0.976	0.138

N: sample sizes; S: number of polymorphic sites; h: number of haplotypes; H: haplotype diversity; π: nucleotide diversity; *bp*: base pairs.

The *BI* tree analysis identified two main clusters of domestic sheep (I and II in Fig 3) supported by high posterior probabilities (Prob ≥ 0.99). Cluster I comprised of the sequences of Cypriot mouflon and sheep *HPG*s C and E, the latter was included into a well-supported sub-cluster.

Cluster II included two well-supported main groups, one of them including *HPG* D, and the other both *HPG*s A and B together with the European mouflon, closely associated to *HPG* B. Among the other *Ovis* species, the most phylogenetically related to Cluster I and II was the urial.

The *ML* analysis (tree not shown) was consistent with *BI*, showing both the same topology and the same highly supported nodes at the main groups retrieved (for the corresponding bootstrap values, see Fig 3).

To quantify the divergence among haplogroups, pairwise genetic distances between groups were calculated under the *K2P* model (S2 Table).

The rate variation among sites was modeled with a gamma distribution (shape parameter = 0.05). The lowest level of genetic differentiation was observed between *HPG* C and E (0.003 ± 0.0005), closely followed by *HPG* A and B (0.005 ± 0.001). The genetic distance between *O*. *g*. *ophion* sequences and the other haplogroups range from 0.005 ± 0.001 (with *HPG* C and E) to 0.011 ± 0.001 (with *HPG* B and D).

The divergence times of splitting events within the genus *Ovis*, resulting from multiple point calibration (see Table 2 for further details), are reported in Table 5 and Fig 3.

**Table 5:** Molecular dating in million years obtained in the present study for the main splitting events within *Ovis* genus based seven calibration points (*CP*).

	BEAST		MEGA		*AV*
	Median	95% *HPD*	Div.time	*CI* 95%	
***Ovis***	2.19	1.52–2.87	3.12	0.158–6.083	2.655
**Argali/Sheep**	1.01	0.69–1.37	1.214	0.059–2.369	1.112
**Urial/Sheep**	0.87	0.58–1.20	0.907	0.043–1.771	0.889
**Domestic sheep**	0.34	0.24–0.46	0.256	0.011–0.501	0.298
***HPG* A + *HPG* B/*HPG* D**	0.26	0.18–0.37	0.21	0.005–0.29	0.235
***HPG* A/*HPG* B**	0.19	0.11–0.27	0.14	0.002–0.42	0.165
***Ovis aries musimon*/*HPG* B**	0.02	0.01–0.04	0.021	-0.007–0.050	0.021
***Ovis gmelini ophion/HPG* C+E**	0.17	0.11–0.24	0.172	0.004–0.340	0.171
***HPG* E/*HPG* C**	0.12	0.07–0.17	0.122	0.000–0.243	0.121
***HPG* B**	0.006	0.007–0.015	0.005	-0.006–0.016	0.006
***HPG* E**	0.03	0.01–0.06	0.033	-0.006–0.071	0.032
***HPG* C**	0.03	0.01–0.05	0.027	-0.006–0.061	0.029
***HPG* D**	0.01	0.001–0.02	0	0–0	0.005
***HPG* A**	0.02	0.01–0.04	0.02	-0.007–0.047	0.020

*AV*: average value.

The ancestor of the genus *Ovis* lived about 2.66 *MYA*. Argali and urial split from domestic sheep 1.11 and 0.89 *MYA*, respectively.

The two main *mtDNA* lineages detected into the domestic sheep radiation (clusters I and II in Fig 3), started 0.3 *MYA*. The split event between the group *HPG* D and the groups *HPGs* A and B, within cluster II, occurred 0.24 *MYA*. *HPGs* A and B diverged from each other about 0.17 *MYA*. Results indicated that the divergence between lineages representing *HPG* B sheep and European mouflon occurred 21 thousand years ago (*KYA*).

Within cluster I, the divergence between *O*. *g*. *ophion* (Cyprus mouflon) and *Ovis* groups *HPGs* C and E occurred about 0.17 *MYA*. *HPGs* C and E diverged from each other about 0.12 *MYA*. The rise of all current haplogroups was estimated to have occurred around 5–35 *KYA*.

### *D-loop* region: features and phylogenetic analyses

A total of 14 complete *D-loops* from two Sardinian and two Chios sheep, two Corsican, three Cypriot and five Sardinian mouflons were here sequenced (*GB*# KR011770-82).

All samples from Sardinia, Corsica and Chios islands harboured a 1,179 *bp* long *D-loop* region, except for one indel occurring in a specimen of Corsican mouflon (1,180). Instead, the three Cypriot mouflons showed a 1,184 *bp* long *D-loop* segment, with four copies of a 76 *bp* long repeat motif, located within a fragment of 304 *bp* long ranging from the 15,654 nt to the 15,957 nt, referring to the whole *mtDNA* domestic sheep sequence (*GB*# NC_001941) [60]. Repeat units of 76 *bp* are typical of sheep belonging to the *HPGs* C and E [12].

As it was already described for *O*. *aries* [60], each repeat contains two octamer sequences of mirror symmetry (TTAATGTA, TACATTAA) which can form stable stem loops. The *D-loop* region exhibits the typical structure with three domains: *ETAS* (extended terminal associated sequences), central and *CSB* (conserved sequence blocks). All blocks of conserved sequences may be identified by comparing them with the respective *O*. *aries* consensus sequence. The *CSB1* has been found at position 16,417–16,444 nt, the *CSB2* + 3 at position 16,477–16,488 nt, whereas a termination-associated sequence (*TAS*-A) was identified at position 16,006–16,020 nt. The marked sequence conservation of both *CSBs* and *TAS* in virtually all mammals supports their hypothesized function [62]. The origin of H-strand replication (*O*_*H*_), and the promoters for H- and L-strand transcription (*HSP*, *LSP*) have also been identified and located at positions 16,398, 16,589 and 16,489 *nt* respectively.

The complete Cyprus mouflon *D-loop* sequence was compared with the homologous sequences of the five sheep *mtDNA* haplogroups identified by Meadows et al. [13] (see Table 1 for details). Three Anatolian mouflon (*O*. *g*. *anatolica*) sequences, representative of each clade (haplogroup A and haplotype X) previously described for this species by Demirci et al. [12], were included into the dataset. The bighorn, the argali and the urial wild sheep sequences were used as outgroups.

Among 35 individuals belonging to seven species within the genus *Ovis*, 23 haplotypes, defined by 645 polymorphic sites (S), were found. Overall high values of total mean haplotype and nucleotide diversity, were obtained, h = 0.976 and π = 0.138, respectively. The Anatolian mouflon (*O*. *g*. *anatolica*) showed the lowest average value of haplotype diversity (h = 0.600), whereas the lowest nucleotide diversity values were found among individuals belonging to the two species *O*. *a*. *musimon* and *O*. *g*. *ophion*. Estimates of genetic diversity for the whole genome are reported in Table 4.

**Fig 4:**
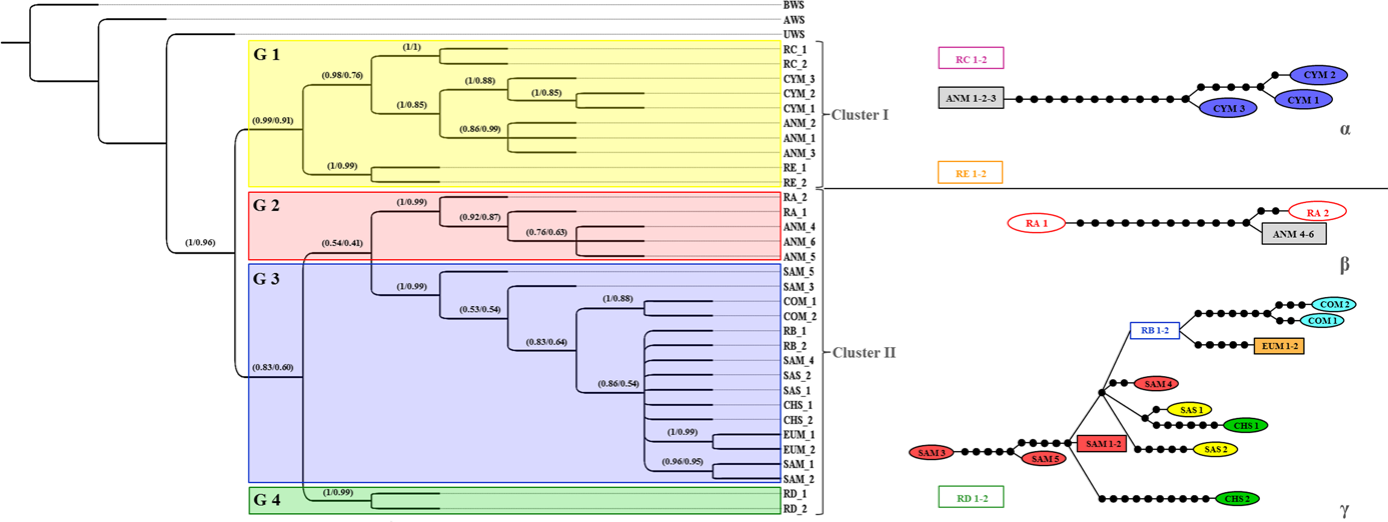
Rooted tree obtained by Bayesian inference for *D-loop* region and the corresponding 95% statistical parsimony networks. Bayesian groups inferred by the Bayesian assignment test are also represented through coloured boxes (G 1 in yellow, G 2 in blue, G 3 in red, G 4 in green). Nodal supports are indicated above the nodes (posterior probability for *BI* / bootstrap values for *ML*). Groups retrieved using the 95% statistical parsimony networks are shown on the right. Each black dot in the networks represents a point mutation. Haplotypes in the network are coloured according to the groups of individuals analyzed (see Table 1 for details). Sample codes are given in Table 1.

*BI* tree analysis identified two different maternal lineages in the radiation of domestic sheep (Fig 4).

Results were consistent with clusters (I and II) identified in the *28H BI* and *ML* tree analysis (Fig 3). Accordingly, cluster I grouped *HPGs* C and E, while cluster II included *HPGs* A, B and D. In accordance with Demirci et al. [12], our results showed that cluster I exclusively grouped individuals with 76 *bp* long repeat units, while cluster II grouped individuals with 75 *bp* long repeat units only.

Within cluster I, two groups were observed. One including the *HPG* E, and the second including the *HPG* C together with some Anatolian and all Cypriot mouflons. In the latter, a well-supported genetic structure was evident between *HPG* C and the mouflons belonging to Anatolia and Cyprus. A further sub-structure was also evident between Anatolian and Cypriot specimens.

The Sardinian, Corsican and European mouflons grouped together within cluster II in a single group along with the domestic sheep from Sardinia and Chios islands and the *HPG* B sequences. Within the same cluster, the remaining Anatolian mouflons are included in a second group along with *HPG* A sequences. The *ML* analysis (tree not shown) was consistent with *BI*, showing both the same topology and the same highly supported nodes at the main groups retrieved (for the corresponding bootstrap values, see Fig 4). Consistently with the *BI* and *ML* tree, Bayesian assignment analysis evidenced the occurrence of four genetic groups hereafter named as G 1 (yellow in Fig 4 and S1 Fig), G 2 (red in Fig 4 and S1 Fig), G 3 (blue in Fig 4 and S1 Fig), G 4 (green in Fig 4 and S1 Fig) (S3 Table; Fig 4 and S1 Fig).

Cluster G 1 included all mouflons from Cyprus, three Anatolian mouflons along with sequences belonging to sheep *HPGs* C and E. Cluster G 2 includes the remaining Anatolian mouflons (three individuals) along with sheep *HPG* A sequences. Cluster G 3 grouped all the mouflons from Sardinia, Corsica, and continental Europe, along with all the domestic sheep from Sardinia and Chios and sequences belonging to sheep *HPG* B. Cluster G 4 included individuals representative of *HPG* D exclusively.

The statistical parsimony network analysis retrieved seven disconnected clusters within the *Ovis* genus. Three main clusters (α, β, γ in Fig 4) encompassed 78% of the individuals, while four additional minor clusters correspond to one or two individuals representative of the domestic sheep *HPGs* C, E and D and of one Anatolian mouflon individual. Cluster α was exclusive of Anatolian and Cypriot mouflons. Cluster β encompassed domestic sheep haplogroup A individuals and some Anatolian mouflons. Cluster γ corresponded to the Bayesian genetic group G 3 above reported.

**Fig 5:**
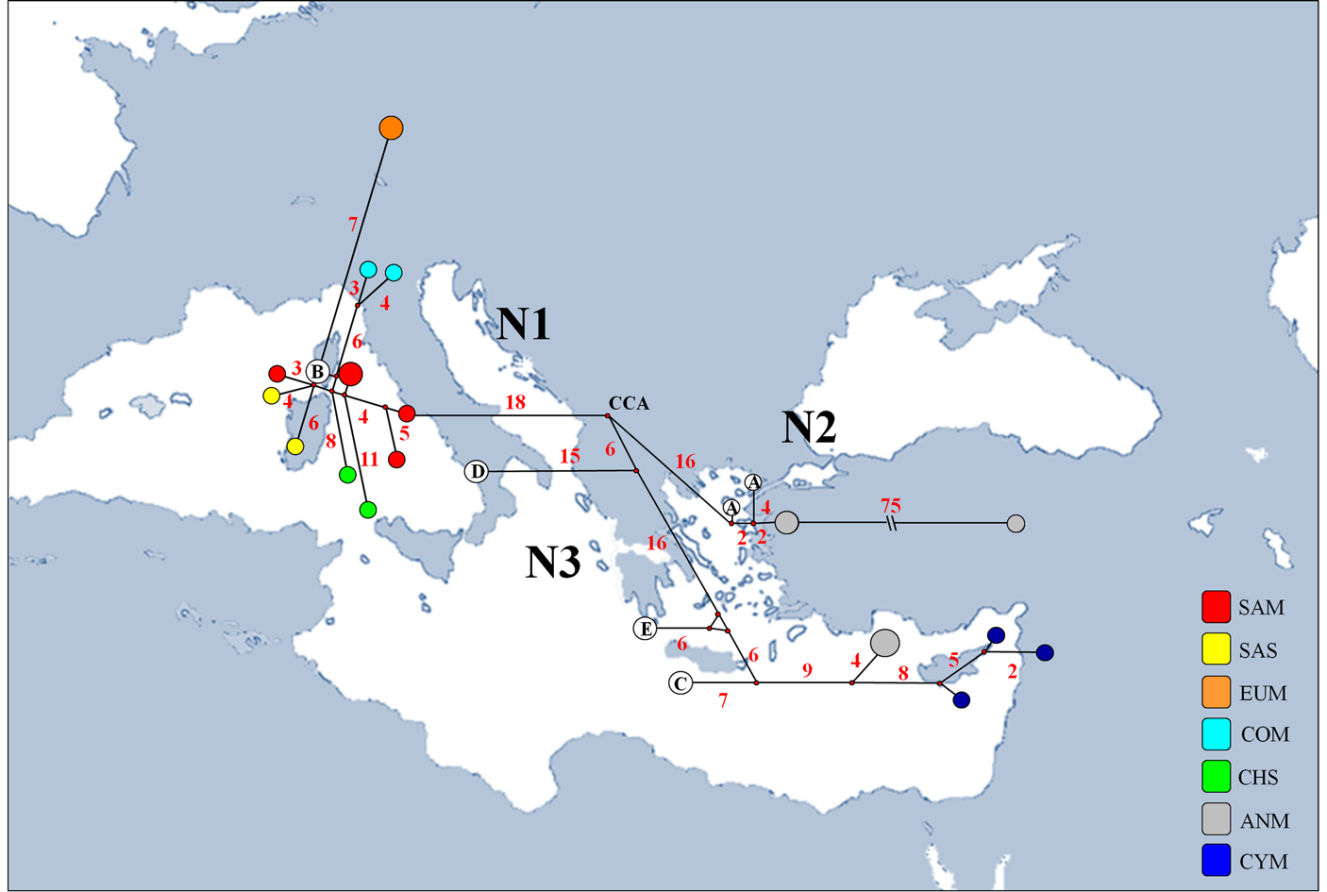
A median-joining network superimposed on the sample map. It highlights the geographic distribution of *D-loop* haplotypes. N1, N2 and N3 indicate the three main groups evidenced in this analysis. *CCA* indicate the likely common ancestor. The capital letters (A, B, C, D and E) inside white spots on the network correspond to the five sheep haplogroups used as references. Small red plots on the nodes, correspond to median vectors representing hypothetic connecting sequences, calculated with a maximum parsimony method. The long branches leading to isolated haplotypes were shortened and indicated with ‘‘\\”. The numbers of mutations between haplotypes that are greater than one are reported on the network branches. Sample codes are given in Table 1.

In accordance with the statistical parsimony network analysis, the median-joining network highlighted the occurrence of three main groups of haplotypes likely sharing a central common ancestor (*CCA*) (Fig 5). A group (N1) exclusively encompassed haplotypes from western Europe and Aegean Sea (Chios) along with individuals representative of the domestic sheep *HPG* B. It corresponds to the Bayesian genetic group G 3 and the statistical parsimony network cluster γ, respectively, and diverged for 18 point mutations from the *CCA*. A further group (N2) encompassed domestic sheep haplogroup A individuals and some Anatolian mouflons. It corresponds to the Bayesian genetic group G 2 and diverged for 16 point mutations from the *CCA*. The last group (N3) encompassed all Cypriot and some Anatolian mouflons along with individuals representative of the domestic sheep *HPGs* C, E and D. It corresponds to the cluster I in *ML* and *BI* tree analysis and diverged for 6 point mutations from the *CCA*. Notably, this group diverged from the *CCA* for a number of point mutations (22) comparable with the other groups if considering only Cypriot and Anatolian mouflons along with *HPGs* C and E. Pairwise genetic distances between groups were calculated using the *K2P* correction model (S4 Table). The lowest level of genetic differentiation was observed between *HPG* C and E (0.018 ± 0.004), closely followed by *HPG* A and B (0.0345 ± 0.006). The genetic distance between Cypriot and Anatolian mouflons carrying the haplotype X was 0.013 ± 0.003. Into cluster II of *ML* and *BI* tree analysis, the average variability between the group composed of Cypriot and Anatolian mouflons carrying the haplotype X and the sheep *HPG* C and E was 0.0235 ± 0.004 and 0.0195 ± 0.0045, respectively. This value is higher than the distance between *HPG* C and E.

## Discussion

The evolution and taxonomy of the genus *Ovis* is still a debated topic, and the relationship between the extant wild sheep and domestic sheep remains unsolved [63]. In the last decades, different wild sheep have been proposed as potential ancestors of domestic sheep, such as the urial, argali and European mouflon. However, based on analyses performed by means of *mtDNA* sequences and endogenous retrovirus [10, 16] none of them can be conclusively confirmed as being the living ancestor of sheep. The Asian mouflon (*O*. *gmelini*) is currently considered as the next closest extant species to domestic sheep [5–7,12].

### Cyprus mouflon mitogenome

In this study the first Asian mouflon mitogenome from a Cypriot individual (*O*. *g*. *ophion*) was provided and compared with those from other *Ovis* species. The *mtDNA* main structural features (see [60–61] for details) did not differ between the Cyprus mouflon and other Caprinae species.

As far as control region variation is concerned, the Cyprus mouflons here analyzed were included in a cluster closely related to the Anatolian individuals carrying the haplotype X [12]. The only mitochondrial haplotype previously reported for *O*. *g*. *ophion* (*HPG* B—[29]) was not detected in this study. However, this may be due to the reduced number of Cypriot sequences analysed here.

### Phylogeography patterns of distribution of sheep haplogroups

Phylogenetic analysis, carried out on the *28H* and *D-loop* regions, evidenced the occurrence of two main phyletic lines emerging from a common ancestor as it was evidenced by *ML* and *BI* tree analysis. Domesticated sheep are included in both clusters thus suggesting the occurrence of a high genetic differentiation among domestic sheep mitochondrial lineages as possible consequence of the domestication of several phylogenetically related ancestors.

The first cluster spread exclusively in the Near East (cluster I in Figs 3 and 4), while the second one spread in the Near East and Europe (cluster II in Figs 3 and 4). Within the Near East cluster, the lowest level of genetic differentiation among haplogroups was detected between *HPGs* C and E, as inferred by genetic distances estimation.

On the basis of *D-loop ML* and *BI* analyses, individuals from Anatolia (mouflons) were included in a cluster (cluster II in Fig 4) grouping sheep *HPGs* A, B and D. Such findings were partially consistent with those provided by Demirci et al. [12]. With the exception of the three Cyprus mouflons, all the *D-loop* sequences obtained in the present study belonged to *HPG* B. The dominance of *HPG* B among European ovine breeds can be explained by the combined action of founder effects and genetic drift, occurred during the migration of the Neolithic farmers into Europe. Otherwise, assuming that the introduction of sheep into Europe may have followed two subsequent waves [16], the *HPG* B dominance could be explained by a massive presence of this lineage among the sheep populations that spread during the second wave.

Analyses performed on the *D-loop* by statistical parsimony network also indicated the presence of an additional well-supported genetic sub-cluster within the *ML* and *BI* cluster I (Fig 4) exclusively grouping the two species of Asian mouflons (*O*. *g*. *ophion* and *O*. *g*. *anatolica*). Genetic distances estimation supports the occurrence of a new haplogroup (*HPG* X). Indeed, as showed in the S4 Table, the distance between *HPGs* C and E (0.018 ± 0.004) is lower than those between *HPG* X and HPG C (0.0235 ± 0.004) or *HPG* X and *HPG* E (0.0195 ± 0.0045). Interestingly, the median-joining network analysis (Fig 5) suggested that sheep *HPG* D could represent the closest haplotype to the past common ancestor for sheep and mouflons.

Results obtained for the *D-loop* region (1,270 bp-long) were not always consistent with those obtained from the whole mitochondrial genome regions (*28H*) (14,426 bp-long). Such a discrepancy may be related to several reasons. Indeed, the *28H* segment is more than ten times longer than the *D-loop* region, which occupies less than 7% of the *mtDNA* genome [64–66]. Furthermore, analysis inferred from the *D-loop* alone can be problematic given that this locus mutates rapidly and it is subjected to saturation due to excessive homoplasy [41, 67]. In addition, several equally likely gene trees can often be inferred from *D-loop* sequences, particularly when large numbers of samples are analyzed [68–69].

### Molecular dating

Based on the results obtained in this study, the group including domestic sheep and Cyprus mouflon diverged from urial around 0.89 *MYA* in accordance with previous analysis of retrotypes and morphological traits which dated this event around 0.8 *MYA* [16]. Furthermore, domestic sheep along with the Cyprus mouflon and urial diverged from argali around 1.11 *MYA*.

The rise of the extant sheep haplogroups is estimated to have occurred at around 5–35 *KYA*, which was about 25 thousand years before the first domestication event, as assumed based on fossil records and archaeozoological evidence [2] (Table 5). Divergence times estimated in this study encompassed the first domestication events suggested by either fossil records and archaeozoological evidence. However, our results do not rule out the chance that first domestication might have occurred earlier than 10 *KYA*.

Our estimates of the earliest radiation of the domestic sheep common ancestor (298 *KYA*) (see Fig 3 for details) are not consistent with Meadows et al. [13], who placed this event around 920 *KYA*. Such a discrepancy might be related to the different molecular marker used by these authors who obtained their estimates on the concatenated sequences of the 12 protein coding genes of the H strand (*12H*), which cover a smaller portion of the whole mitogenome if compared with the *28H* segment used in the present study.

The coalescence estimates provided by Meadows et al. [13] might also be affected by the use of a single *Cyt B* gene-based calibration point not directly referred to a fossil reference. The use of a smaller molecular marker combined with the use of only one calibration point may affect the estimation of divergence times. However, we should underline that such a discrepancy might as well reflect the different method used to estimate this event. In fact, estimates obtained using APE, assuming a relaxed clock with correlated rates, were similar to those obtained by Meadows et al. [13] (0.89 versus 0.92 *MYA*). Noteworthy, the method used in the present study for estimating divergence times in APE is partly based on the Penalized Likelihood (data not shown). Based on our molecular dating, the five sheep haplogroups here retrieved originated between 5–35 *KYA*. This is consistent with a sympatric or allopatric differentiation event likely occurring in the Near East among wild sheep populations during the Pleistocene. In such a context, the current sheep haplogroups would not be the result of multiple, independent domestication events but rather represent the remnants of an ancient high genetic variability which spread to Asia and Europe during the Neolithic human migrations [30]. Indeed, it is estimated that goat and sheep domestication events occurred around 10.5–11 *KYA* in regions encompassing northern Zagros (North of Iraq) and south eastern Anatolia (South-East Turkey) [1, 2, 5, 25–26, 70]. The coalescence estimates here provided for the rise of the Cypriot mouflon (190 *KYA*) set this event considerably earlier than the origin of sheep haplogroups (10–30 *KYA*).

The island of Cyprus is considered one of the first areas where domesticated sheep were introduced [16, 25], thus offering a valuable insight into the evolution of its unique endemic mouflon. At the present time, the most probable hypothesis is that mouflons were brought to Cyprus by humans about 12 *KYA* [12], following an independent evolutionary history, most likely facilitated by the absence of potential predators/competitors as supported by fossil records [71–72].

However, an alternative hypothesis that large mammals, including mouflons, have seemingly arrived on Cyprus before the arrival of humans is also possible. During the period of minimum sea levels through Pleistocene glacial maxima, the sea dropped to at least 125 m below the current level, and the Cyprus shoreline expanded towards the mainland by several kilometres (see Fig 1 from [25] for more details). It has also been reported that between 25 and 18 *KYA* there were small mounts above sea level, forming three islands between Cyprus and the mainland that might have been used by animals as a stepping stone pathway for an average period of 10 *KYA* [25]. Cyprus Pleistocene fossil sites consist almost exclusively of pygmy hippopotamus (*Phanouirios minutus*) and pygmy elephant (*Elephas cypriotes*) [71–72]. The lack of sheep fossils could be consistent with the absence of mouflons and other wild sheep in the island during this period [71]. However, it is worth noting that the sheep’s preference for mountainous habitats and the relative unfavourable conditions for fossil preservation [73] might have prevented the finding of fossil records.

Even considering such an alternative hypothesis relating to the early presence of mouflon in Cyprus, it appears more likely that the high genetic distance observed between the Cypriot mouflon and other sheep haplogroups is due to a limited crossbreeding with domestic sheep. Indeed, contrary to what happened in the other main Mediterranean islands of Corsica and Sardinia, sheep farming has never been crucial to the economy of Cyprus, where for a long period the “fat tailed” sheep was the only variety of domestic sheep on the island until the introduction of new breeds from Israel and Europe in the 1970s [74–76].

Fat-tail breeds are an important class of sheep breeds characterized by specific phenotypic traits, that are first documented as being present 5,000 *YA* [77–78]. These breeds are well adapted to arid regions thanks to the fat deposition stored in the tail that represent an energy reserve during times of drought and feed shortage. The fat-tail sheep are commonly found in a wide geographical range with subtropical to semi-arid climate, especially including the Middle East and North Africa [78]. The fact that the fat-tail breeds are now prevalent in the Fertile Crescent, where sheep were originally domesticated, while thin-tail sheep breeds are predominant in peripheral areas [78], and that the wild ancestor of sheep is thin-tail suggests that the first domesticated sheep had a thin-tail and the fat-tail was developed later [77].

These findings suggest that the Cypriot mouflon, which never experienced modern selection strategies, can be considered as a relic of the first wild domesticated populations [12, 16] likely representing one of the closest descendants of the Palaeolithic Anatolian wild sheep. As a consequence of its geographic isolation, it presumably returned early to a feral or semi-feral state before the secondary event of domestication occurred in South-West Asia, involving in a second time Europe, Africa and the rest of Asia [16]. Some Cypriot primitive sheep might have survived the migrations of the secondary domesticated breeds from South-West Asia in areas without predators or by occupying sites less involved in trading contacts [16]. Future phylogeographic analyses on a larger number of Cypriot mouflons might be useful in depicting the population dynamics which affected the origin of the species on the island to better clarify the relationships between *O*. *g*. *ophion* and other ovine breeds, such as the Cypriot fat-tail sheep, thus providing useful insights into the management of the Cypriot distinct genetic conservation unit.

The threefold molecular dating methodologies used in this study enabled us to provide a well-supported estimate of the time of coalescence of the current ovine haplogroups setting their origin between 5–35 *KYA*. These lineages probably extended their range towards Europe only at around the 10-11^th^ millennium *BP*, when human populations moved to the continent from the Near East [79–80].

In conclusion, the Cyprus mouflon whole *mtDNA* sequence here provided improves the panel described by Meadows *et al*. [13] thus identifying a potential new sheep haplogroup which includes the Cypriot and Anatolian mouflons. However, since the whole *mtDNA* sequence of the Anatolian mouflon is not available, this hypothesis was inferred by the analysis performed on the *D-loop* sequencing data only and requires to be confirmed by means of deeper analyses on the whole mitogenome. Additionally, further analysis on a larger number of samples from Near East and Europe might shed light on the occurrence of new haplogroups never described before, resulting in a more complete overview of the phylogenetic relationships among ovine breeds from different geographic areas.

Finally, the molecular data provided in this study may also play an important role in functional genomics or functional pathways related to energy metabolism. Indeed, *mtDNA* have an important role in bioenergy production and thermogenesis and thus in climate adaptation [81–82]. Genomic research can provide additional knowledge on thermal impact effects on the energy metabolism. In such a context, the analysis of the Cyprus mouflon whole mitogenome sequence could be useful to identify mutations potentially related to mitochondrial heat production [82], improving the knowledge on the adaptation to a subtropical to semi arid climate similar to the climate of Cyprus. These new genetic tools will enable researchers to review the history of the species and the geographic distribution of haplogroups in the light of environmental change adaptation that occurred during evolution, and to increase the efficiency of breed traits selection related to the reproductive ability in semi-arid areas [83] in order to improve food security.

## Supporting Information

S1 Fig Bayesian clustering analysis for *D-loop* region. Estimated genetic structure in the dataset analyzed as inferred using the Bayesian model-based clustering analysis. Each individual is represented by a thin vertical line colored according to its belonging to one of the four clusters retrieved. Black lines separate individuals from different sampling sites. Sample codes are listed in Table 1.
(TIF)

S1 Table List of the primers used to carry out *PCR* and sequencing reactions to obtain the first Cyprus mouflon *mtDNA* sequence. *bp*: base pairs; Ta: annealing temperature. In the Forward and Reverse columns, numbers in parenthesis refer to the position of the 3’ end L-strand.
(PDF)

S2 Table Estimates of evolutionary divergence between whole genome (*28H*) sequences. Genetic distances are shown below the diagonal and standard deviations above the diagonal. Analyses were conducted using the *K2P* model. Sample codes are listed in Table 1.
(PDF)

S3 Table Frequencies distributions of the four Bayesian genetic groups found in the present study for *D-loop* region. N: sample size; %: relative frequency of distribution. Sample codes are listed in Table 1.
(PDF)

S4 Table Estimates of evolutionary divergence between *D-loop* sequences. The genetic distances are shown below the diagonal and standard deviations above the diagonal. Analyses were conducted using the *K2P* model. Sample codes are listed in Table 1.
(PDF)
