# Supplementary figures and images for: The First Mitogenome of the Cyprus Mouflon (Ovis gmelini ophion): New Insights into the Phylogeny of the Genus Ovis
Source: PLoS One. 2015 Dec 4;10(12):e0144257. doi: 10.1371/journal.pone.0144257 (PMC4670089; doi:10.1371/journal.pone.0144257)

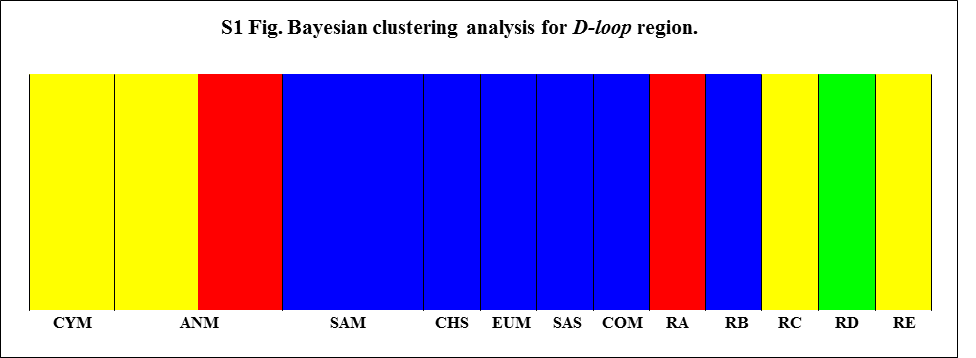

Supplement: S1 Fig — Estimated genetic structure in the dataset analyzed as inferred using the Bayesian model-based clustering analysis. Each individual is represented by a thin vertical line colored according to its belonging to one of the four clusters retrieved. Black lines separate individuals from different sampling sites. Sample codes are listed in Table 1. (TIF) [file pone.0144257.s001.tif]
