# Supplementary material for: The First Mitogenome of the Cyprus Mouflon (Ovis gmelini ophion): New Insights into the Phylogeny of the Genus Ovis
Source: PLoS One. 2015 Dec 4;10(12):e0144257. doi: 10.1371/journal.pone.0144257 (PMC4670089; doi:10.1371/journal.pone.0144257)
Supplement: S1 Table — bp: base pairs; Ta: annealing temperature. In the Forward and Reverse columns, numbers in parenthesis refer to the position of the 3’ end L-strand. (PDF) [file pone.0144257.s002.pdf]

S1 Table. List of the primers used to carry out *PCR* and sequencing reactions to obtain the first Cyprus mouflon *mtDNA* sequence.

Primer pairs for standard PCR					
Primer code	Forward*	Reverse*	bp	Ta °C	Contigs overlapping (bp)
CL1-CH1	cccccccttccccttaaatatt (16,497)	tttgctgaagatggcggtat (699)	843	60	177
CL2-CH2	agagtactaccggcaacagc (574)	tttcatcattcccttgcggt (1,235)	681	52	145
CL3-CH3	cacgtaaagatgttaggtcaa (754)	ttgttgatttatccccgcct (2,154)	1,421	52	502
CL4-CH4	tctgaccgtgcaaaggta (2,034)	tagggttagtgctaggatggg (2,988)	974	52	100
CL5-CH5	ttctcctagctgtagcttttc (2,803)	ggtctaggttgatggatggaa (4,367)	1,585	55	164
CL6-CH6	tgctcatctgaattggattcg (4,010)	tggttgtggttgagttggcta (4,589)	600	62	378
CL7-CH7	caggcctaatcttactcacatg (4,308)	gcatgtgcggttacaattacg (5,517)	1,231	52	303
CL8-CH8	caaccaaccacaaagatatcg (5,378)	cctccgactgtgaaaagaaag (6,387)	1,030	57	160
CL9-CH9	acgggcttacttcacgtcag (6,254)	aaggatggaagagcaatcat (7,261)	1,027	52	153
CL10-CH10	gtcaagattaaattacaggtg (7,000)	tagggaatagtaagctagga (8,033)	1,054	52	282
CL11-CH11	ttttcaactaaaaatctca (7,862)	taagtgggtgaatgcctgtt (8,989)	1,146	55	190
CL12-CH12	aaaaaggtcttcactacggaa (8,863)	tgtgagggatactgtgaatgc (9,936)	1,094	60	147
CL13-CH13	acgaatgaacccaaaaaggac (9,800)	caaggaccatggagcctgcaa (10,869)	1,090	60	157
CL14-CH14	tttacacactagcaggatccc (10,647)	ttatttcttgtcctgtgtgga (11,937)	1,311	60	243
L11715-H13606	ttaggaaccaaaaaattggtgcaa (11,738)	attggttattgttactggttggt (13,630)	1,916	64	223
CL15-CH15	tatccaccacaatcacaagcc (13,491)	ggagagagccaaagtttcatc (14,267)	797	60	160
L14110-H15323	gaccaatgatatgaaaaaccatcg (14,133)	gttctccttctctggtttacaaga (15,352)	1,243	54	158
CL16-CH16	agccagttgaacacccctaca (15,200)	tgttgggtttatgaacgctc (15,617)	438	62	173
CR1-CR2	cccactatcaacacccaaagct (15,411)	tcatctaggcattttcagtgcctt (48)	1,279	58	228

Primer pairs for nested PCR					
Primer code	Forward*	Reverse*	bp	Ta °C	Fragment
N1-N2	ggttgagcctccaactcaaaa (3,123)	ctcgtgggttgtgctttttt (4,064)	962	52	CL5-CH5
N3-N4	ttattcttgtcactgcaaaca (12,159)	tttgggggattgttattggg (13,191)	1,053	60	L11715-H13606

Primers exclusively used for sequencing			
Primer code	Sequence*	Sequencing direction	Fragment
HS1	tttttggtaaacaggcgggg (1,945)	Counterclockwise	CL3-CH3
LS1	tattaatatccctaatcc (8,165)	Clockwise	CL11-CH11
LS2	aaactccaactatgaacg (11,182)	Clockwise	CL14-CH14
LS3	taccttcctagaaacatgaaa (14,499)	Clockwise	L14110-H15323
HS2	tcattaaatagttacccc (16,147)	Counterclockwise	CR1-CR2
