# Supplementary material for: The First Mitogenome of the Cyprus Mouflon (Ovis gmelini ophion): New Insights into the Phylogeny of the Genus Ovis
Source: PLoS One. 2015 Dec 4;10(12):e0144257. doi: 10.1371/journal.pone.0144257 (PMC4670089; doi:10.1371/journal.pone.0144257)
Supplement: S2 Table — Genetic distances are shown below the diagonal and standard deviations above the diagonal. Analyses were conducted using the K2P model. Sample codes are listed in Table 1. (PDF) [file pone.0144257.s003.pdf]

S2 Table. Estimates of evolutionary divergence between whole genome (*28H*) sequences.

	RD_1	RD_2	RB_1	RB_2	EUM	RA_1	RA_2	RC_1	RC_2	RE_1	RE_2	CYM
RD_1		0,000	0,001	0,001	0,001	0,001	0,001	0,001	0,001	0,001	0,001	0,001
RD_2	0,000		0,001	0,001	0,001	0,001	0,001	0,001	0,001	0,001	0,001	0,001
RB_1	0,007	0,007		0,000	0,000	0,001	0,001	0,001	0,001	0,001	0,001	0,001
RB_2	0,007	0,008	0,000		0,000	0,001	0,001	0,001	0,001	0,001	0,001	0,001
EUM	0,007	0,008	0,001	0,000		0,001	0,001	0,001	0,001	0,001	0,001	0,001
RA_1	0,007	0,007	0,005	0,005	0,005		0,000	0,001	0,001	0,001	0,001	0,001
RA_2	0,006	0,007	0,005	0,005	0,005	0,000		0,001	0,001	0,001	0,001	0,001
RC_1	0,010	0,010	0,011	0,011	0,011	0,010	0,009		0,000	0,001	0,001	0,001
RC_2	0,010	0,011	0,011	0,011	0,011	0,010	0,010	0,001		0,000	0,000	0,001
RE_1	0,010	0,010	0,010	0,010	0,010	0,009	0,009	0,003	0,003		0,000	0,001
RE_2	0,010	0,011	0,010	0,010	0,010	0,010	0,009	0,003	0,003	0,001		0,001
CYM	0,011	0,011	0,011	0,011	0,011	0,010	0,010	0,005	0,005	0,005	0,005	
