# Supplementary material for: The First Mitogenome of the Cyprus Mouflon (Ovis gmelini ophion): New Insights into the Phylogeny of the Genus Ovis
Source: PLoS One. 2015 Dec 4;10(12):e0144257. doi: 10.1371/journal.pone.0144257 (PMC4670089; doi:10.1371/journal.pone.0144257)
Supplement: S3 Table — N: sample size; %: relative frequency of distribution. Sample codes are listed in Table 1. (PDF) [file pone.0144257.s004.pdf]

S3 Table. Frequencies distributions of the four Bayesian genetic groups found in the present study for *D-loop* region.

Cluster	Specimens	N	%
G 1	ANM_4, ANM_5, ANM_6, RA_1, RA_2	5	15.6
G 2	RD_1, RD_2	2	6.3
G 3	CHS_1, CHS_2, COM_1, COM_2, EUM_1, EUM_2, RB_1, RB_2, SAM_1, SAM_2, SAM_3, SAM_4, SAM_5, SAS_1, SAS_2	15	46.9
G 4	RE_1, RE_2, RC_1, RC_2, CYM_1, CYM_2, CYM_3, ANM_1, ANM_2, ANM_3	10	31.2
