# Supplementary material for: The First Mitogenome of the Cyprus Mouflon (Ovis gmelini ophion): New Insights into the Phylogeny of the Genus Ovis
Source: PLoS One. 2015 Dec 4;10(12):e0144257. doi: 10.1371/journal.pone.0144257 (PMC4670089; doi:10.1371/journal.pone.0144257)
Supplement: S4 Table — The genetic distances are shown below the diagonal and standard deviations above the diagonal. Analyses were conducted using the K2P model. Sample codes are listed in Table 1. (PDF) [file pone.0144257.s005.pdf]

S4 Table. Estimates of evolutionary divergence between *D-loop* sequences.

	CYM_2	CYM_1	CYM_3	ANM_3	ANM_2	ANM_1	RB_1	RB_2	RE_1	RE_2	RC_1	RC_2	RD_1	RD_2	RA_1	RA_2	ANM_5	ANM_4	ANM_6
CYM_2		0.001	0.002	0.003	0.003	0.003	0.006	0.006	0.005	0.005	0.004	0.004	0.006	0.006	0.006	0.006	0.006	0.006	0.006
CYM_1	0.001		0.002	0.004	0.004	0.004	0.006	0.006	0.005	0.005	0.004	0.004	0.006	0.006	0.006	0.006	0.006	0.006	0.006
CYM_3	0.006	0.007		0.003	0.003	0.003	0.006	0.006	0.005	0.005	0.004	0.004	0.006	0.006	0.006	0.006	0.006	0.006	0.006
ANM_3	0.014	0.015	0.010		0.000	0.000	0.006	0.006	0.004	0.004	0.004	0.004	0.006	0.006	0.006	0.006	0.006	0.006	0.006
ANM_2	0.014	0.015	0.010	0.000		0.000	0.006	0.006	0.004	0.004	0.004	0.004	0.006	0.006	0.006	0.006	0.006	0.006	0.006
ANM_1	0.014	0.015	0.010	0.000	0.000		0.006	0.006	0.004	0.004	0.004	0.004	0.006	0.006	0.006	0.006	0.006	0.006	0.006
RB_1	0.036	0.035	0.042	0.041	0.041	0.041		0.000	0.006	0.006	0.006	0.006	0.006	0.006	0.006	0.006	0.006	0.006	0.006
RB_2	0.036	0.035	0.042	0.041	0.041	0.041	0.000		0.006	0.006	0.006	0.006	0.006	0.006	0.006	0.006	0.006	0.006	0.006
RE_1	0.027	0.028	0.025	0.020	0.020	0.020	0.034	0.034		0.000	0.004	0.004	0.006	0.006	0.006	0.005	0.006	0.006	0.006
RE_2	0.027	0.028	0.025	0.020	0.020	0.020	0.034	0.034	0.000		0.004	0.004	0.006	0.006	0.006	0.005	0.006	0.006	0.006
RC_1	0.022	0.023	0.020	0.017	0.017	0.017	0.039	0.039	0.018	0.018		0.000	0.006	0.006	0.007	0.006	0.006	0.006	0.006
RC_2	0.022	0.023	0.020	0.017	0.017	0.017	0.039	0.039	0.018	0.018	0.000		0.006	0.006	0.007	0.006	0.006	0.006	0.006
RD_1	0.037	0.038	0.041	0.038	0.038	0.038	0.034	0.034	0.032	0.032	0.036	0.036		0.000	0.006	0.006	0.006	0.006	0.006
RD_2	0.037	0.038	0.041	0.038	0.038	0.038	0.034	0.034	0.032	0.032	0.036	0.036	0.000		0.006	0.006	0.006	0.006	0.006
RA_1	0.042	0.041	0.046	0.043	0.043	0.043	0.037	0.037	0.038	0.038	0.043	0.043	0.038	0.038		0.002	0.002	0.002	0.002
RA_2	0.039	0.038	0.043	0.040	0.040	0.040	0.032	0.032	0.033	0.033	0.040	0.040	0.034	0.034	0.005		0.002	0.002	0.002
ANM_5	0.040	0.039	0.044	0.041	0.041	0.041	0.035	0.035	0.036	0.036	0.041	0.041	0.034	0.034	0.004	0.003		0.000	0.000
ANM_4	0.040	0.039	0.044	0.041	0.041	0.041	0.035	0.035	0.036	0.036	0.041	0.041	0.034	0.034	0.004	0.003	0.000		0.000
ANM_6	0.040	0.039	0.044	0.041	0.041	0.041	0.035	0.035	0.036	0.036	0.041	0.041	0.034	0.034	0.004	0.003	0.000	0.000	
